# Loss of Cadherin-11 in pancreatic ductal adenocarcinoma alters tumor-immune microenvironment

**DOI:** 10.3389/fonc.2023.1286861

**Published:** 2023-10-26

**Authors:** Aimy Sebastian, Kelly A. Martin, Ivana Peran, Nicholas R. Hum, Nicole F. Leon, Beheshta Amiri, Stephen P. Wilson, Matthew A. Coleman, Elizabeth K. Wheeler, Stephen W. Byers, Gabriela G. Loots

**Affiliations:** ^1^ Lawrence Livermore National Laboratory, Physical and Life Science Directorate, Livermore, CA, United States; ^2^ Georgetown-Lombardi Comprehensive Cancer Center, Department of Oncology, Georgetown University Medical Center, Washington, DC, United States; ^3^ University of California Davis Health, Department of Orthopaedic Surgery, Sacramento, CA, United States

**Keywords:** PDAC, CDH11, CAF, IL33, MDSC, T cells

## Abstract

Pancreatic ductal adenocarcinoma (PDAC) is one of the top five deadliest forms of cancer with very few treatment options. The 5-year survival rate for PDAC is 10% following diagnosis. Cadherin 11 (Cdh11), a cell-to-cell adhesion molecule, has been suggested to promote tumor growth and immunosuppression in PDAC, and Cdh11 inhibition significantly extended survival in mice with PDAC. However, the mechanisms by which Cdh11 deficiency influences PDAC progression and anti-tumor immune responses have yet to be fully elucidated. To investigate *Cdh11*-deficiency induced changes in PDAC tumor microenvironment (TME), we crossed *p48-Cre; LSL-Kras^G12D/+^; LSL-Trp53^R172H/+^* (KPC) mice with *Cdh11^+/-^* mice and performed single-cell RNA sequencing (scRNA-seq) of the non-immune (CD45^-^) and immune (CD45^+^) compartment of KPC tumor-bearing *Cdh11* proficient (*KPC-Cdh11^+/+^*) and *Cdh11* deficient (*KPC-Cdh11^+/-^*) mice. Our analysis showed that *Cdh11* is expressed primarily in cancer-associated fibroblasts (CAFs) and at low levels in epithelial cells undergoing epithelial-to-mesenchymal transition (EMT). *Cdh11* deficiency altered the molecular profile of CAFs, leading to a decrease in the expression of myofibroblast markers such as *Acta2* and *Tagln* and cytokines such as *Il6*, *Il33* and Midkine *(Mdk)*. We also observed a significant decrease in the presence of monocytes/macrophages and neutrophils in *KPC-Cdh11^+/-^* tumors while the proportion of T cells was increased. Additionally, myeloid lineage cells from *Cdh11*-deficient tumors had reduced expression of immunosuppressive cytokines that have previously been shown to play a role in immune suppression. In summary, our data suggests that *Cdh11* deficiency significantly alters the fibroblast and immune microenvironments and contributes to the reduction of immunosuppressive cytokines, leading to an increase in anti-tumor immunity and enhanced survival.

## 1 Introduction

Pancreatic ductal adenocarcinoma (PDAC) is one of the deadliest types of cancer with a limited survival rate of ~10% over five years (1). In general, by the time a patient presents with symptoms, the disease has advanced to a surgically unresectable stage and likely metastasized to other vital organs leading to rapid mortality (2). Since conventional chemotherapy (FOLFIRINOX, gemcitabine, gemcitabine + abaraxane, etc.) prolongs patients’ lives for only a few months (3), it is necessary to identify new therapies or better therapeutic targets.

Our immune system, when activated, can elicit an antitumor response that can have long-term clinical benefits and could contribute to prolonged survival. Infiltration of immune cells into the tumor microenvironment (TME) has been associated with various disease prognoses depending on the type of immune cells present and has been leveraged to improve patient survival through immunotherapy in several types of cancers (4, 5). For example, T cells are conventionally the focus of already approved immunotherapies (6–9), and B cells show great promise for future immunotherapies as high B cell infiltration correlates with better survival in PDAC patients (10). As compared to “hot” tumors with inflammation and infiltrated T cells, PDACs are considered to be immunologically “cold” with low levels of tumor-infiltrating lymphocytes, which presents a challenge to established immunotherapies (11–13). A deeper understanding of PDAC immunobiology is necessary to make PDACs amenable to immune-based therapies.

PDAC tends to be surrounded by cells that suppress anti-tumor immune responses. Major immune suppressive cells in the TME include tumor-associated macrophages (TAMs), myeloid-derived suppressor cells (MDSCs), tumor-associated neutrophils (TANs) and regulatory T cells (Treg) (14). These immunosuppressive cells hinder CD4 and CD8 T cell response as well as the ability of natural killer (NK) and antigen-presenting cells (APC) to exert effective tumor surveillance, consequently leading to an inhibition of the anti-tumor immune responses (15). Cancer associated fibroblasts (CAFs) are another major cell type in the TME that can contribute to immunosuppression (14). CAFs are a vastly heterogeneous cell population and are the most prominent stromal cell type in pancreatic cancer (16). CAFs promote tumor proliferation, invasion and metastasis by secreting various growth factors and cytokines and by modifying the tumor extracellular matrix (ECM) (14, 17, 18). In addition, CAFs contribute to an immunosuppressive microenvironment through secretion of multiple cytokines and chemokines and reciprocal interactions with immune cells that mediate the recruitment and functional differentiation of these cells (16). A deeper understanding of the PDAC TME, specifically the coordinated actions of tumor supporting immune cells and CAFs against lymphocytes is necessary to make immune-based therapies feasible for effectively treating PDAC.

Cadherin 11 (Cdh11) is a mechanosensitive transmembrane protein involved in cell adhesion (19, 20) and plays a role in WNT signaling by modulating β-catenin (21, 22). In PDAC, it is primarily expressed by CAFs, and it was recently shown that *Cdh11* deficiency induces antitumor immunity, reduces immunosuppression, and increases survival in tumor bearing mice (23). Furthermore, the administration of a small molecule inhibitor blocking Cdh11 increased the efficacy of gemcitabine, a common anti-cancer chemotherapeutic (23). However, the Cdh11 inhibitor, was ineffective in reducing tumor burden of mT3 pancreatic tumor bearing immunosuppressed *Rag1*-mutant mice, suggesting that T and B cells are required for immunomodulation of PDAC mediated by Cdh11 inhibition (23). Also, *Cdh11* deficiency induced immune memory in *Cdh11^-/-^* mice that cleared tumors; these mice did not form new tumors upon subsequent re-challenges with the same cancer cells (23). The cellular and molecular mechanisms behind *Cdh11* deficiency-induced antitumor activity and its relationship to immunosuppression are not yet fully understood.

Understanding how *Cdh11* promotes an immunosuppressive tumor microenvironment in PDAC will provide invaluable insights into developing new clinical approaches for effective eradication of cancer cells, in solid tumors that are classically immunodeficient. Using an established genetically engineered PDAC mouse model (GEMM) (*p48-Cre;LSL-Kras^G12D/+^;LSL-Trp53^R172H/+^*) that has a median survival of 5 months (known as KPC mice) (24), we investigated *Cdh11*-deficiency induced changes in PDAC immune microenvironment. Using single-cell RNA sequencing (scRNA-seq) we compared the tumor microenvironment of *Cdh11*-deficient (KPC-*Cdh11^+/-^)* and wildtype *(KPC-Cdh11^+/+^)* mice with pancreatic tumors and identified immune subpopulations that correlate with decreased tumor burden and improved survival. Our study suggests that *Cdh11* deficiency alters the molecular profiles of CAFs resulting in decreased expression of immunosuppressive cytokines within the TME. We also observed an increase in T cell infiltration and a loss of TAMs/MDSCs and neutrophils in the tumor of *KPC-Cdh11^+/-^* mice and identified several genes differentially expressed in these populations as a result of *Cdh11*-deficency. Knowledge of immune cell subtypes and genes found as altered in the TME as a result of a *Cdh11-*deficiency and their relationship to tumor prognosis will provide a basis for further development of novel therapies for PDAC.

## 2 Methods

### 2.1 Animal husbandry

*Cdh11^-/-^* (https://www.jax.org/strain/023494) (25) and KPC (*p48-Cre;LSL-Kras^G12D/+^;LSL-Trp53^R172H/+^*) mice were bred to each other to generate cohorts of *KPC-Cdh11^+/+^*, and *KPC-Cdh11^+/-^* as previously described (23). Mice were housed in a pathogen-free environment under standard conditions at Georgetown University (GU) and Lawrence Livermore National Laboratory (LLNL). All animal work was conducted under approved Institutional Animal Care and Use Committee (IACUC) protocols at GU and LLNL and conformed to the National Institute of Health (NIH) guide for the care and use of laboratory animals.

### 2.2 Pancreas collection and single-cell isolation

*KPC-Cdh11^+/+^*, *KPC-Cdh11^+/-^* and *Cdh11^+/+^* cohorts (n≥4, mixed sex) were euthanized at 4-5 months of age (23). Pancreases were excised and single-cell suspensions were prepared using a collagenase digestion solution (3 mg/ml Collagenase I (ThermoFisher Scientific Catalog #17018029), 100 µg/mL DNase I (Roche Catalog #11284932001), Dispase II (Roche Catalog #4942078001) in RPMI1640 media supplemented with 10% FBS (ThermoFisher Scientific, Catalog #11875101) for 1 hour with shaking at 37°C. Digests were filtered through a 100 µm cell strainer and depleted of red blood cells using ACK lysis buffer, per manufacturer guidelines (Gibco, Catalog #A1049201). Remaining cells were analyzed for viability and CD45 expression. CD45^-^/7-AAD^-^ (non-immune) and CD45^+^/7-AAD^-^ (immune) cells populations were sorted *via* fluorescently activated cell sorting (FACS) and collected for scRNA-seq preparation.

### 2.3 FACS analysis

Cells from digested pancreases were stained in a 1:100 dilution of APC/Cyanine7 anti-mouse CD45 (Biolegend, Catalog #103116) in PBS with 1% FBS and incubated for 20 minutes in the dark. Cells were resuspended in 7-AAD Viability Staining Solution (BioLegend, Catalog #420404) and analyzed on a BD FACSMelody cytometer, sorting CD45^+^/7-AAD^-^ and CD45^+^/7-AAD^-^ populations for scRNA-seq analysis.

### 2.4 scRNA-seq and data analysis

Single-cell libraries were prepared from sorted cell populations from *KPC-Cdh11^+/+^*, *KPC-Cdh11^+/-^* and *Cdh11^+/-^* mice using the Chromium Single Cell 3′ GEM, Library & Gel Bead Kit v3 (10x Genomics, Pleasanton, CA, USA; catalog no. 1000075) on a 10x Genomics Chromium Controller following manufacturers protocol and sequenced using an Illumina (San Diego, CA, USA) NextSeq 500 sequencer as described before (26). The scRNA-seq data was demultiplexed and aligned against mouse reference genome mm10 using Cell Ranger Single-Cell Software Suite (10x Genomics, Pleasanton, CA, USA) to obtain Unique Molecular Identifier (UMI) counts. Subsequent analysis was performed in Seurat (27) as described before (26). Briefly, after pre-processing, normalization, feature selection and data scaling, data from various experimental groups were integrated to generate an integrated dataset. Subsequently, dimensional reduction by principal component analysis (PCA), clustering, Uniform Manifold Approximation and Projection (UMAP) reduction, and visualization of clusters were performed in Seurat as described before (26). Genes differentially expressed between clusters were identified using ‘FindMarkers’ function implemented in Seurat. For each experimental group, the cell type proportions were estimated as a ratio of the number of cells in each cell cluster relative to the total number of cells sequenced. Any CD45^+^ clusters found in non-immune scRNA-seq data or CD45^-^ clusters found in immune data were excluded from the analysis.

### 2.5 Analysis of human PDAC samples

Human PDAC scRNA-seq data from early and metastatic tumors (28) were obtained from the Gene Expression Omnibus database (GSE205013). The raw barcode, feature, and matrix were downloaded and analyzed using Seurat (29), as described above to identify the cell types and cell type-specific gene expression as outlined in the original analysis (28). Correlation between CDH11 expression and immune cell infiltration was determined using TIMER (http://timer.cistrome.org/). Correlation between the expression of CDH11 and other genes in human tumors was determined using TNMplot (https://tnmplot.com/). UALCAN (https://ualcan.path.uab.edu) was used to determine protein expression of CDH11 in PDAC patient tumors, using data from Clinical Proteomic Tumor Analysis Consortium (CPTAC).

### 2.6 Immunohistochemical analysis

Tissues were embedded and sectioned as previously described (23). Formalin-Fixed Paraffin-Embedded (FFPE) 5µm thick sections were deparaffinized with xylene and rehydrated through a series of decreasing ethanol concentrations. Heat induced antigen retrieval method (HIER) using either sodium citrate (pH 6) at 65 °C or universal antigen retrieval solution (Unitrieve) at 65°C for 45 minutes was used to expose antigens for immunohistochemical staining. Slides were counterstained with DAPI and stained for proteins of interest using the following primary and secondary antibodies: Pdgfra (Abcam, ab203491), Cdh11 (ThermoFisher 32-1700), Il33 (ThermoFisher, PA5-47007), CD8a (Abcam, ab21769), F4/80 (Abcam, ab111101) and Ly6g (Abcam, ab238132), anti-rabbit IgG Alexa Fluor 488 (Catalog #A21441, ThermoFisher Scientific), anti-rat IgG2a Alexa Fluor 488 (Catalog #A11006, ThermoFisher Scientific) and anti-rabbit IgG Alexa Fluor 594 (Catalog #A11037, ThermoFisher Scientific). IL33, F4/80 and Ly6g IHC staining was quantified using ImageJ (https://imagej.nih.gov/ij/). Total cell numbers were estimated based on DAPI nuclear staining and cell types of interest were identified as cells with positive signal for IL33, F4/80 or Ly6g that colocalized with the nuclear DAPI stain. Cells exhibiting positive signal were then quantitated relative to the total number of cells within an image. CD8a expression was quantified with a Vectra quantitative pathology imaging system and Inform software.

### 2.7 Cytokine array analysis

Sera and pancreases collected at the time of euthanasia from *KPC-Cdh11^+/+^* and *KPC-Cdh11^+/-^* mice were analyzed for cytokine expression using Mouse XL Cytokine Array (R&D Systems). Equal amount of protein lysates or serum belonging to the same experimental group were pooled together before analysis. This data has been previously described (23).

### 2.8 Statistical analysis

Two-proportion Z-test was used to identify statistically significant differences between cell proportions. A *p* value <0.05 was considered as significant.

## 3 Results

### 3.1 Cdh11 deficiency alters CAF profile in PDAC

**Figure 1:**
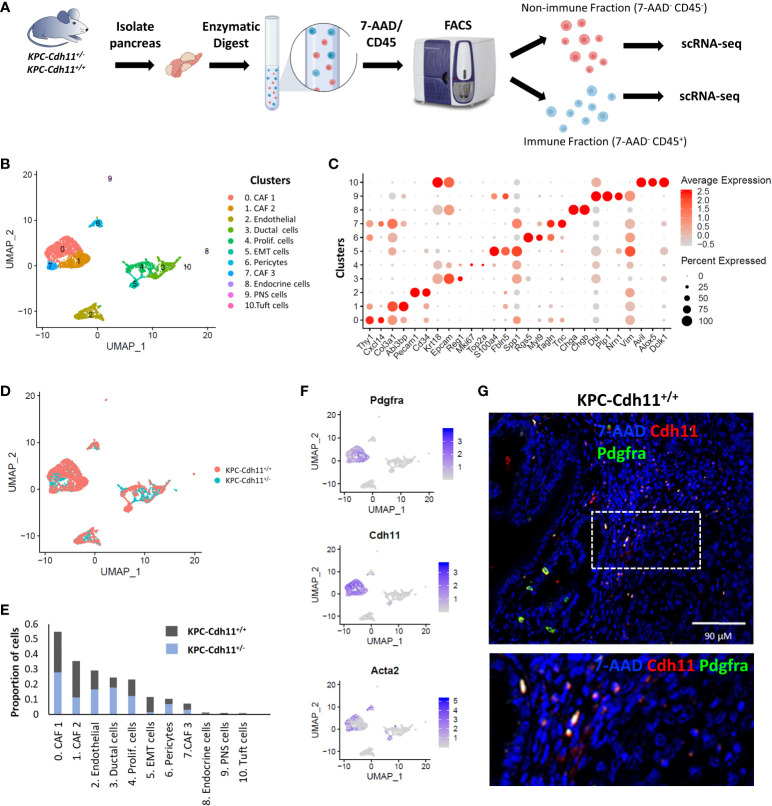
Single-cell level analysis of Cdh11 deficiency-induced changes in stromal cells. **(A)** Experimental design. CD45^+^ (immune) and CD45^-^ (non-immune) cells from pancreases of KPC-Cdh11^+/+^ and KPC-Cdh11^+/-^ mice were isolated and both fractions were analyzed separately using scRNA-seq. **(B)** Non-immune cell clusters from both KPC-Cdh11^+/+^ and KPC-Cdh11^+/-^ mice visualized by Uniform Manifold Approximation and Projection (UMAP). Each color represents a cell type/subtype with distinct transcriptomic profiles. **(C)** Dot plot showing the expression of cell type markers corresponding to the cell clusters shown in panel **(A)**. Dot size represents the fraction of cells expressing a gene in a cluster and intensity of color indicates the average expression level in that cluster. **(D)** UMAP plot colored by experimental condition. **(E)** Graph showing the proportion of various cell types in each experimental group, calculated using scRNA-seq data. **(F)** Feature plot showing the enrichment of Pdgfra, Cdh11 and myofibroblast marker Acta2 in CAF clusters. **(G)** IHC showing co-expression of Cdh11 and Pdgfra in KPC-Cdh11^+/+^ mice. The dotted boxed area is shown on the bottom with higher magnification.

To investigate the role of Cdh11 in PDAC, we isolated non-immune (CD45^-^) and immune (CD45^+^) cells from pancreases of *KPC-Cdh11^+/+^* and *KPC-Cdh11^+/-^* mice and both fractions were analyzed separately using scRNA-seq (**Figure 1A**). First, non-immune scRNA-seq data was computationally analyzed to determine *Cdh11* deficiency-induced changes in cancer and stromal cells. CD45^-^ cells were clustered using an unbiased clustering approach which identified eleven clusters with distinct gene expression profiles (**Figures 1B-D**). The identity of each cluster was determined based on the expression of previously established cell type-specific markers (**Figures 1B, C**) (23, 26, 30, 31).

CAFs were one of the most abundant cell types identified in pancreatic cancer TME and robustly expressed markers such as *Pdgfra*, *Col3a1* and *Dcn* (**Figures 1C, F**). Based on scRNA-seq data, *KPC-Cdh11^+/+^* mice had ~22.6% more CAFs in their TME compared to *KPC-Cdh11^+/-^* mice (Two-proportion z-test; *p* value <0.00001). Consistent with Cdh11 known expression in CAFs, *Cdh11* expression was primarily observed in CAF clusters, which also had robust expression of CAF marker *Pdgfra* (**Figure 1F**). Further IHC analysis showed co-expression of *Pdgfra* and *Cdh11* in *KPC-Cdh11^+/+^* mice, confirming that CAFs in PDAC robustly express Cdh11 (**Figure 1G**, **S1**). Analysis of pancreases from wildtype mice without tumors (*Cdh11^+/+^*) revealed that normal fibroblasts also express *Cdh11*, suggesting that *Cdh11* may play a significant role in healthy resident fibroblasts as well (**Figure S2**). Low level of *Cdh11* expression was also observed in cells undergoing epithelial-to-mesenchymal transition (EMT) in PDAC (**Figure S3A**). Interestingly, *KPC-Cdh11^+/+^* mice had significantly more EMT cells than *KPC-Cdh11^+/-^* mice (**Figure 1E**). We also analyzed previously published human ‘early’ and ‘metastatic’ PDAC scRNA-seq data (28) and found that CDH2^+^ EMT cells expressed low levels of *CDH11* (**Figure S3B-C**). However, in human tumors also CAFs were the predominant *CDH11*-expressing cells (**Figure S3C**).

**Figure 2:**
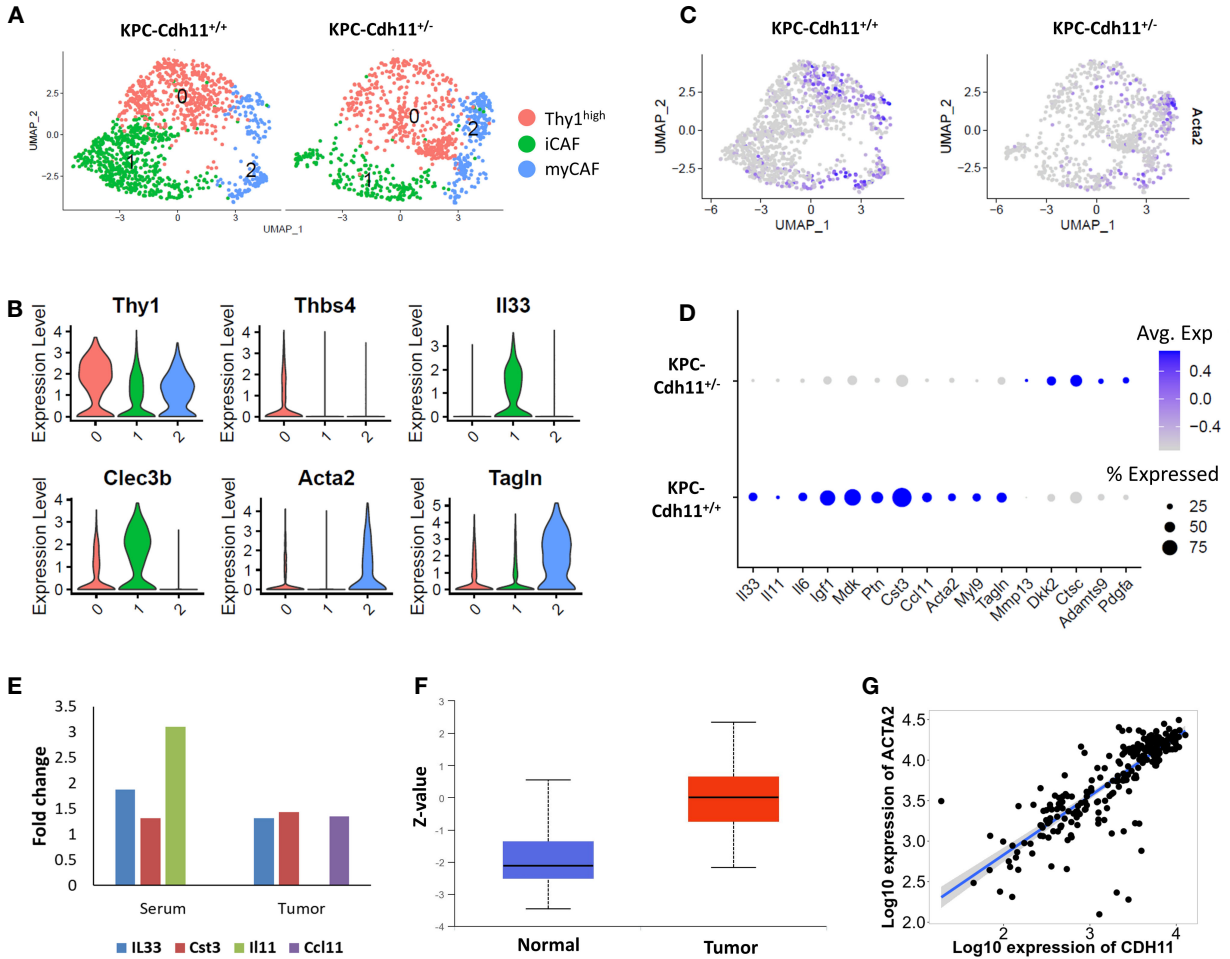
Comparative analysis of KPC-Cdh11^+/+^ and KPC-Cdh11^+/-^ derived CAFs. **(A)** UMAP plot showing various CAF subtypes identified in KPC-Cdh11^+/+^ and KPC-Cdh11^+/-^ mice. **(B)** Violin plot showing the expression of selected CAF subtype markers in scRNA-seq data from both KPC-Cdh11^+/+^ and KPC-Cdh11^+/-^ tumors. **(C)** Feature plot showing the expression of myofibroblast marker Acta2 in each experimental group. **(D)** Dot plot showing a subset of CAF genes differentially expressed between KPC-Cdh11^+/-^ and KPC-Cdh11^+/+^ mice. **(E)** Cytokine array analysis showing fold increase in the expression of various cytokines in serum and tumor samples from KPC-Cdh11^+/+^ mice compared to KPC-Cdh11^+/-^ mice. **(F)** CDH11 protein expression in human pancreatic cancer (CPTAC data from UALCAN). Z-values (Y-axis) represent standard deviations from the median protein expression across samples. **(G)** Correlation between the expression of CDH11 and ACTA2 genes in human PDAC.

**Figure 3:**
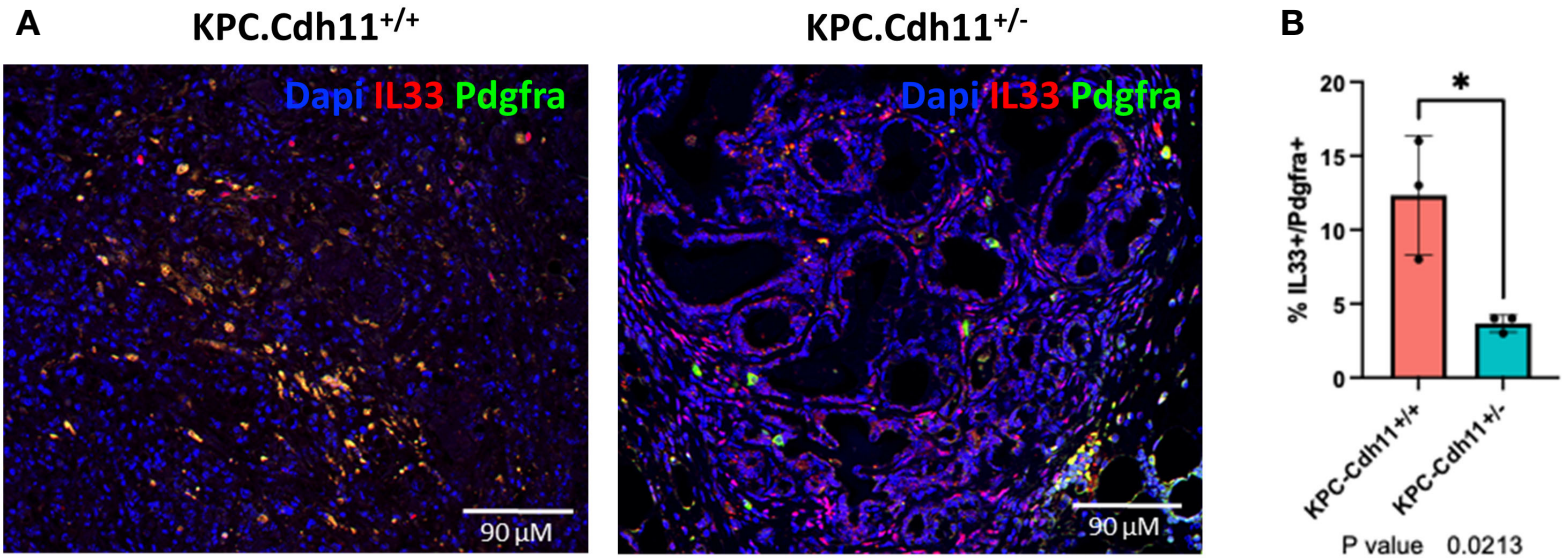
Il33 expression in CAFs. **(A)** IHC analysis showed an increased number of Il33-expressing CAFs (Pdgfra^+^ cells) in KPC-Cdh11^+/+^ pancreas compared to KPC-Cdh11^+/-^ pancreas. **(B)** IHC quantification of Il33 in CAFs (Pdgfra^+^ cells) from KPC-Cdh11^+/+^ and KPC-Cdh11^+/-^ pancreases. *P value <0.05.

To further understand *Cdh11* deficiency-induced changes in CAFs, we extracted cells from CAF clusters (cluster 0, 1 and 7; **Figure 1B**) and analyzed them in more detail. This analysis identified three CAF subtypes including *Acta2* ^high^ myofibroblasts (myCAFs), *Il33* ^high^ inflammatory CAFs (iCAFs), and a *Thy1* ^high^ fibroblast cluster (**Figures 2A-C**). Consistent with our previous findings (23), CAFs from *KPC-Cdh11^+/+^* mice expressed higher levels of *Acta2* than *KPC-Cdh11^+/-^* mice (**Figure 2D**). Expression of other myofibroblast markers such as *Tagln* and *Myl9* were also elevated in *KPC-Cdh11^+/+^* mice (**Figure 2D**). We also identified several other genes differentially expressed between *KPC-Cdh11^+/+^* and *KPC-Cdh11^+/-^* derived CAFs. These genes included cystatin C (*Cst3*), inflammatory cytokines *Ccl11*, *Il33*, *Il11* and *Il6*, insulin growth factor 1 (*Igf1*) and heparin binding growth factors midkine (*Mdk*) and pleiotrophin (*Ptn*), all of which had higher expression in *KPC-Cdh11^+/+^* mice. Proteases *Adamts9* and *Mmp13* and Wnt inhibitor *Dkk2* were found to be upregulated in *KPC-Cdh11^+/-^* mice (**Figure 2D**). Furthermore, a cytokine array analysis showed increased Il33 and Cst3 expression in both tumor and serum from *KPC-Cdh11^+/+^* mice compared to *KPC-Cdh11^+/-^* mice, while Il11 was highly enriched in *KPC-Cdh11^+/+^* serum alone and Ccl11 was enriched in tumor alone (**Figure 2E**). Il33 is a member of the IL-1 family of cytokines, secreted by a variety of cells including epithelial, endothelial and fibroblast-like cells (32). It has been suggested that the increased expression of IL33 in CAFs promotes tumor growth and metastasis *via* modulation of the immune system (32). Consistent with the cytokine data, IHC confirmed a higher number of Il33-expressing CAFs in *KPC-Cdh11^+/+^* mice (**Figure 3**, **S4**). *Il11*, *Il6*, *Ccl11*, *Mdk* and *Ptn* have also been shown to play key roles in cancer progression and immune modulation (33–38), suggesting that altered expression of these genes in *KPC-Cdh11^+/-^* mice may contribute to changes in tumor immune profile. Igf1 signaling has also been implicated in tumor growth, metastasis, drug resistance in PDAC (39).

Next, we analyzed publicly available human pancreatic cancer data (data from CPTAC) and found that CDH11 protein expression was significantly elevated in tumor samples compared to normal (**Figure 2F**). Additionally, we observed a strong positive correlation between the expressions of *CDH11* and *ACTA2* genes in human PDAC (**Figure 2G**). Consistent with our scRNA-seq data, genes such as *IL33* and *IGF1* also showed a positive correlation with *CDH11* expression while *PDGFA* was negatively correlated (**Figure S5**). Together, these findings further confirm the role of Cdh11 in cancer progression and suggest that *Cdh11* deficiency in stromal cells may significantly alter the TME.

### 3.2 Cdh11-deficient stroma promotes immune infiltration in KPC tumors

**Figure 4:**
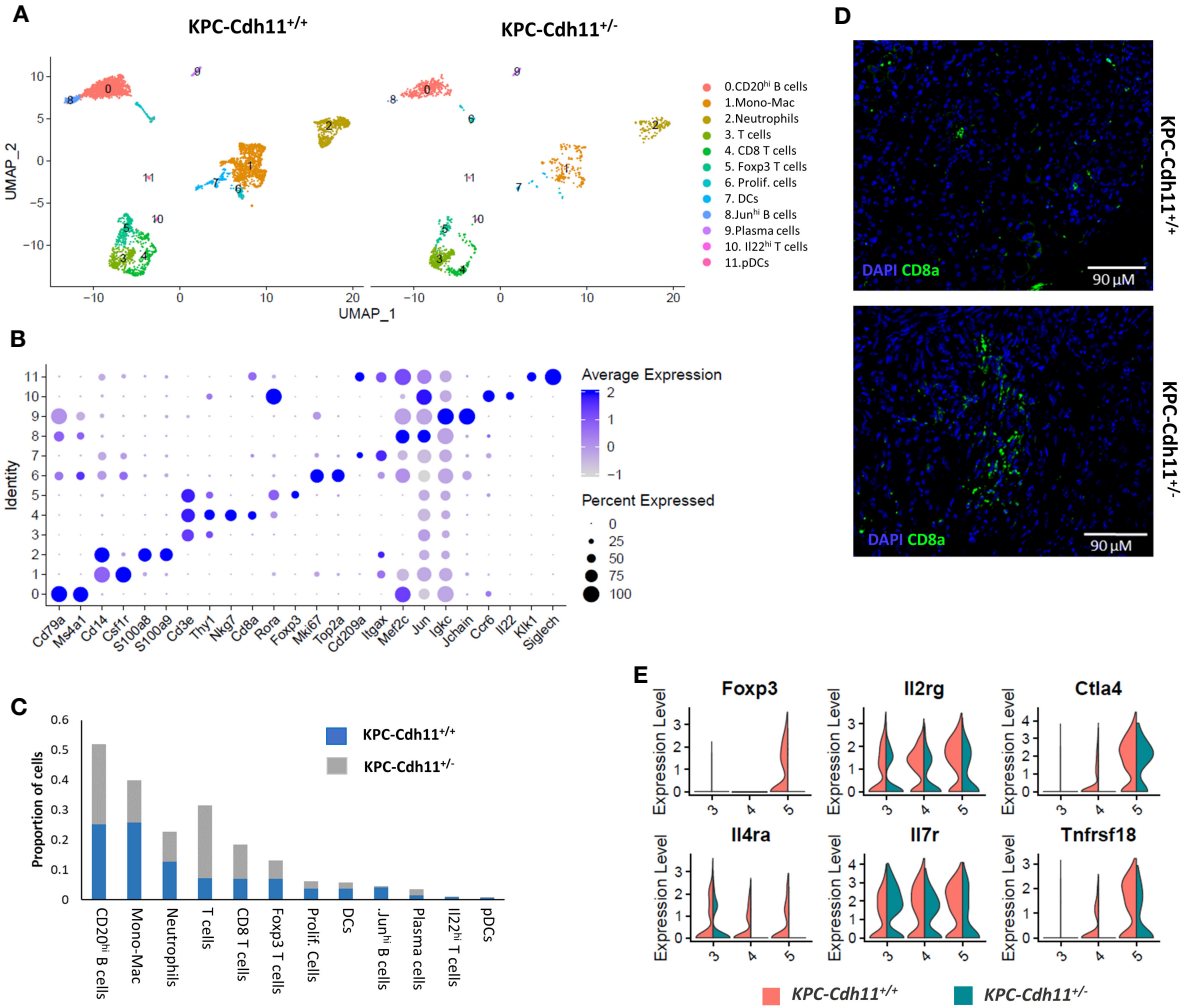
Characterization of pancreas-derived immune cells. **(A)** Immune cell clusters from KPC-Cdh11^+/+^ and KPC-Cdh11^+/-^ mice visualized by UMAP. **(B)** Dot plot showing the expression of immune cell markers in scRNA-seq data from both KPC-Cdh11^+/+^ and KPC-Cdh11^+/-^ mice. Dot size signifies the percentage of cells in that cluster that express a particular gene, while strength of color denotes average expression in that cluster. **(C)** Bar graph showing the proportion of each cell type in both experimental groups, calculated from the scRNA-seq data. **(D)** IHC showing CD8 T cell infiltration in tumors. **(E)** Violin plot showing the differential expression of selected T cell genes in various T cell clusters.

Next, we investigated how the lack of Cdh11 in the stroma impacts the tumor immune microenvironment. CD45-expressing cells purified and quantified by FACS were found in higher proportion in the *KPC-Cdh11^+/-^* than in the *KPC-Cdh11^+/+^* pancreas (**Figure S6A**), suggesting an increase in immune infiltration when the Cdh11 is absent from the PDAC stroma. scRNA-seq analyses of these CD45^+^ cells from *KPC-Cdh11^+/+^* and *KPC-Cdh11^+/-^* mice identified twelve distinct immune cell subtypes including macrophages, neutrophils, dendritic cells (DCs), plasmacytoid dendritic cells (pDCs), B cells and T cells (**Figure 4A**). Cells in cluster 0 had high expression levels for members of the B cell receptor (BCR) signaling complex: *Cd79a* and *Cd79b* (40). This cluster also expressed *Ms4a1* (CD20) and was identified as CD20^hi^ B cells (**Figure 4B**). Cluster 8 also expressed moderate levels of *Cd79a* and *Cd79b*, in addition to showing enrichment for *Jun* and *Mef2c*. This cluster was identified as Jun^hi^ B cells. Cluster 1 had high transcript levels of monocyte/macrophage genes *Cd14* and *Csf1r*, designating this grouping of cells as monocytes/macrophages (Mono-Mac) (41) (**Figure 4B**). Genes critical to T cell signaling including *Thy1*, *Cd3e* and *Cd3d* were highly expressed in clusters 3, 4, 5 and 10 (42). Cluster 5 had additional markers including *Foxp3* and *Rora* and was identified as Foxp3 T cells while cluster 4 was identified as Cd8 T cells based on the expression of cytotoxic T cell markers *Cd8a* and *Nkg7* (**Figure 4B**). Cluster 10 represented a small proportion of cells expressing high levels of *Il22* and *Ccr6* and was identified as Il22^hi^ T cells. Cluster 2 was identified as neutrophils based on high expression of *S100a8* and *S100a9* and cluster 6 was annotated as proliferating cells based on the expression of *Mki67* and *Top2a* (**Figures 4A, B**). Cluster 7 expressed DC markers while cluster 11 expressed markers of pDCs. *Jchain* and *Igkc*, two genes highly expressed in plasma cells were enriched in cluster 9 (43–46).

Consistent with our previous finding (23), T cells and Cd8 T cells were found in high abundance in scRNA-seq data from *KPC-Cdh11^+/-^* tumors (*Two-proportion z-test; p<0.05).* (**Figure 4C**). IHC analysis further confirmed increased Cd8 T cell infiltration in *KPC-Cdh11^+/-^* tumors (**Figure 4D**, **S6B-C**). We also found that *KPC-Cdh1^+/+^* tumors had a slightly higher proportion of FOXP3+ T cells compared to *KPC-Cdh11^+/-^ (P value*: 0.18). In addition, we observed elevated expression of *FoxP3*, interleukin 2 receptor gamma (*Il2rg*), *Il4ra*, *Il7r*, cytotoxic T-lymphocyte-associated protein 4 (*Ctla4*), and glucocorticoid-induced tumor necrosis factor receptor-related protein (*Tnfrsf18*) in *KPC-Cdh1^+/+^* tumors (**Figure 4E**), many of which have been previously suggested to play critical roles in Treg differentiation or function (23, 47–49). We also observed a small (not statistically significant) increase in the proportion of Cd20^hi^ B cells in *KPC-Cdh11^+/-^* mice while Jun^hi^ B cells were significantly reduced.

### 3.3 Myeloid infiltration is decreased in Cdh11-deficient tumors

**Figure 5:**
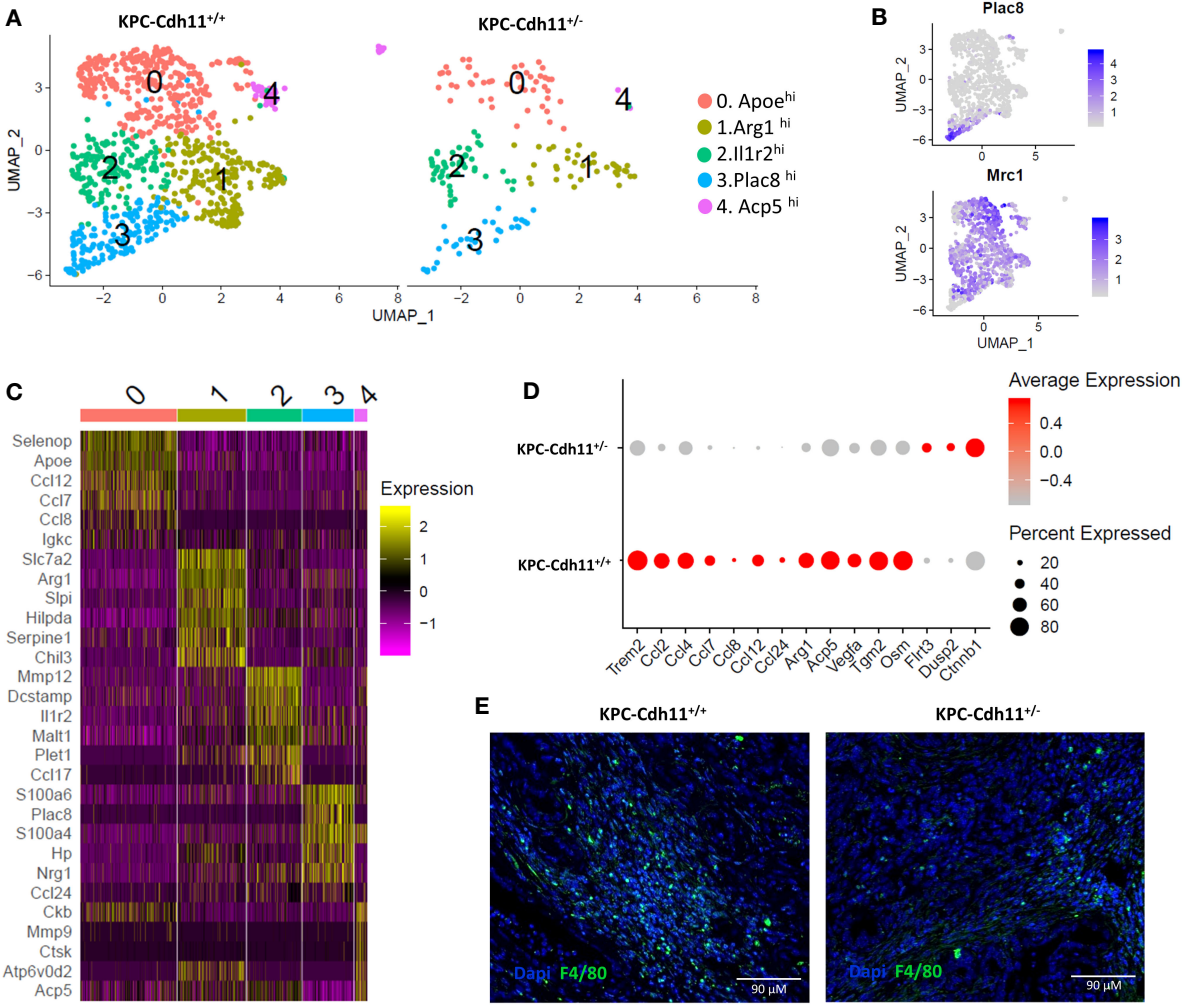
Monocytes and macrophages from pancreases of KPC-Cdh11^+/-^ and KPC-Cdh11^+/+^ mice. **(A)** UMAP plot showing subtypes of Mono-Mac cells across mice of different Cdh11 status. **(B)** Feature plots denoting expression of Mrc1 and Plac8. **(C)** Heatmap showing expression of selected immune signaling molecules enriched in clusters 0-4. **(D)** Dot plot showing differential expression of cytokines and other selected genes in Mono-Macs from KPC-Cdh11^+/-^ and KPC-Cdh11^+/+^ mice. **(E)** IHC showing F4/80 expressing macrophages in KPC-Cdh11^+/-^ and KPC-Cdh11^+/+^ mice.

The monocyte/macrophage population was dramatically reduced in *KPC*-*Cdh11^+/-^* mice compared to *KPC*-*Cdh11^+/+^* mice (*Two-proportion z-test; p<0.00001)* (**Figure 4C**). To further analyze differences in monocyte and macrophage subpopulations between *KPC*-*Cdh11^+/+^* and *KPC*-*Cdh11^+/-^*, all cells from the Mono-Mac cluster (cluster 1; **Figure 4A**) were extracted and re-analyzed using Seurat which resulted in five different groups with distinct transcriptional profiles (**Figure 5A**). Cluster 3 expressed monocyte markers such as *Plac8* and *Ly6c2* while all other clusters highly expressed tumor associated macrophage (TAM) marker *Mrc1* and *Trem2* (**Figure 5B**, **S7A**). However, these macrophage-like clusters had distinct gene expression profiles. Cluster 0 showed enrichment for *Apoe* and several cytokines including *Ccl7*, *Ccl8* and *Ccl12* whereas cluster 1 highly expressed markers of M2-like polarization including *Arg1* and *Chil3* (**Figure 5C**). Cluster 2 showed enrichment for *Il1r2*, *Mmp12* and *Dcstamp* while cluster 4 expressed osteoclasts markers including *Acp5*, *Ctsk* and *Mmp9* and likely represent osteoclast-like giant-cells (50). We also observed that monocytes and macrophages from *KPC*-*Cdh11^+/+^* mice expressed higher levels of chemokines including *Ccl2*, *Ccl4*, *Ccl6*, *Ccl7*, *Ccl8* and *Osm* compared to *KPC*-*Cdh11^+/-^* mice (**Figure 5D**). Increased expression of TAM marker Trem2 (51) was also observed in *KPC*-*Cdh11^+/+^* mice (**Figure 5D**). Furthermore, IHC analysis showed that *KPC*-*Cdh11^+/+^* mice had more F4/80 expressing TAMs than *KPC*-*Cdh11^+/-^* mice (**Figure 5E**, **S7B-C**).

**Figure 6:**
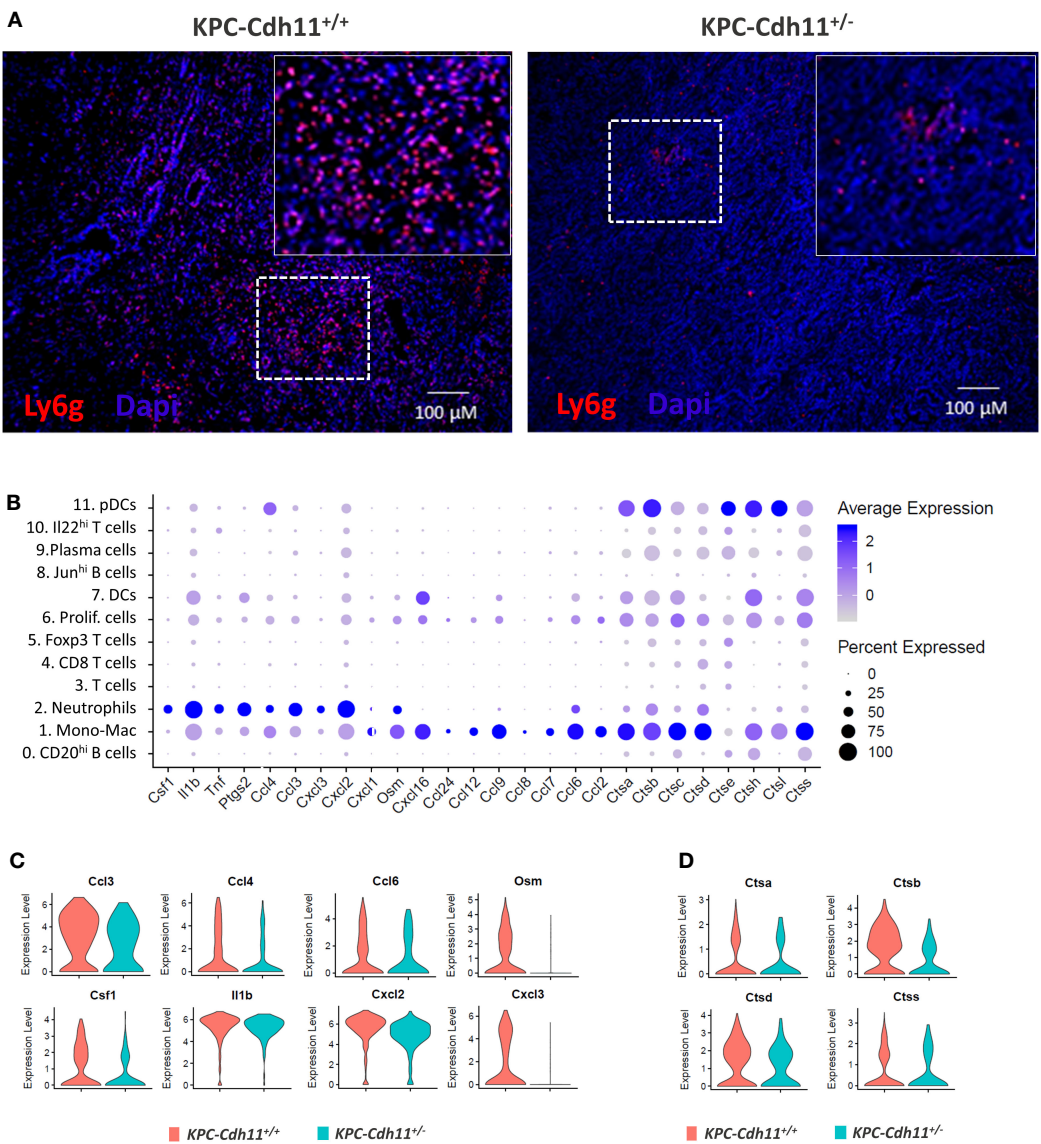
Neutrophils from pancreases of KPC-Cdh11^+/-^ and KPC-Cdh11^+/+^ mice. **(A)** IHC analysis showing increased neutrophil infiltration in KPC-Cdh11^+/+^ mice. The dotted boxed area is shown on the right with higher magnification. **(B)** Dot plot showing cytokine expression in various immune cell clusters from both KPC-Cdh11^+/-^ and KPC-Cdh11^+/+^ tumors. Dot size signifies the percentage of cells in each immune cluster that express a particular gene, while strength of color denotes average expression in that cluster. **(C)** Violin plots showing a subset of immune modulatory cytokine genes differentially expressed between neutrophils from KPC-Cdh11^+/-^ and KPC-Cdh11^+/+^ mice. **(D)** Increased expression of cathepsins in neutrophils from KPC-Cdh11^+/+^ mice compared to KPC-Cdh11^+/+^ mice.

Neutrophils were another major immune cell type identified in the PDAC TME and the proportion of neutrophils was reduced in *KPC*-*Cdh11^+/-^* mice compared to *KPC*-*Cdh11^+/+^* mice (*Two-proportion z-test; p <0.01)* (**Figure 4C**, **6A**, **S8A-B**). Compared to other immune cell clusters, neutrophils showed significant enrichment for inflammatory genes *Il1b*, *Tnf*, *Ptgs2*, *Cxcl2*, *Cxcl3*, and *Csf1*, a cytokine involved in macrophage recruitment and differentiation (**Figure 6B**). Neutrophils also expressed additional cytokines and chemokines including oncostatin M (*Osm*), *Ccl6* and *Cxcl1* and proteases such as Cathepsin A (*Ctsa), Ctsb, Ctsc* and *Ctsc*, although Mono-Mac cluster had the highest expression values for these genes (**Figure 6B**). We observed that neutrophils from *KPC*-*Cdh11^+/-^* mice had lower levels of *Csf1* compared to *KPC*-*Cdh11^+/+^* mice, which may have contributed to decreased number of TAMs in these mice (**Figure 6C**). In addition, cytokines and chemokines including *Osm*, *Cxcl2*, *Cxcl3*, *Ccl3*, *Ccl4*, *Ccl6* and *Il1b* were also downregulated in neutrophils from *KPC*-*Cdh11^+/-^* mice (**Figure 6C** *)*. Cathepsins including *Ctsa*, *Ctsb* and *Ctsd* were also significantly downregulated in neutrophils from *Cdh11-*deficient mice (**Figure 6D**). In addition to Mono-Macs and neutrophils, proportion of DCs was also reduced in *KPC*-*Cdh11^+/-^* mice (**Figure 4C**). Consistent with these results, we found a strong positive correlation between CDH11 expression and neutrophil and myeloid DC infiltration in human PDAC (Rho > 65, P <0.0001; **Figure S8C**), suggesting that reduced CDH11 expression in PDAC may contribute to reduced myeloid infiltration to the TME.

A survey of The Cancer Genome Atlas (TCGA) suggests that *CDH11* expression is also elevated in many other cancers including breast cancer (BRCA), cholangiocarcinoma (CHOL), colon adenocarcinoma (COAD), esophageal carcinoma (ESCA), head and neck cancer (HNSC), stomach adenocarcinoma (STAD) and glioblastoma (GBM) (**Figure S9**). It has previously been suggested that increased *CDH11* expression indicates a poor prognosis in advanced gastric cancer (52) and triple negative breast cancer (TNBC) (22). These findings suggest that Cdh11 plays a role in multiple cancer types and Cdh11 inhibition may promote survival in these cancers.

## 4 Discussion

Here we describe several key cellular and molecular changes in stromal and immune cell populations associated with the loss of *Cdh11* in PDAC. Particularly, we show significant changes in CAFs in response to the Cdh11 deficiency. In both human and mouse PDAC, CDH11 was primarily expressed by CAFs. CAFs from *KPC*-*Cdh11^+/-^* mice had significantly lower expression of myofibroblast markers and several immune modulatory factors including *Il6*, *Il11*, *Il33*, *Ccl11*, *Mdk* and *Ptn*. Consistent with this, we observed a reduction in monocyte, macrophage, DC and neutrophil infiltration in these Cdh11-deficient tumors while the proportion of T cells increased. This suggests that *Cdh11* deficiency in CAFs may alter the tumor’s immune profile.

While changes in infiltrating T cell populations have been previously observed as a result of *Cdh11* deficiency (23), here we further highlight CD8^+^ T cells to be correlative with C*dh11* deficient tumors. These cytotoxic T cells may be directly responsible for the reduced tumor burden, and enhanced response to gemcitabine treatment and extended survival of *KPC-Cdh11^+/-^* mice relative to *KPC- Cdh11^+/+^* (23). Consistent with previously reported findings by Peran et al., we also observed a significant decrease in the expression of genes such as *Foxp3*, *Il2rg*, *Il4ra*, *Ctla4* and *Tnfrsf18* that play a role in differentiation, activation or function of Tregs (47–49, 53), in *KPC-Cdh11^+/-^* pancreases (**Figure 4E**).

MDSCs in tumors have been identified to block the recruitment of anti-tumor T/NK cells (54, 55). Reduction in myeloid cells (**Figure 4C**) associated with Cdh11 deficiency may have contributed to increased T cell infiltration and enhanced survival observed in these mice (23). Furthermore, several chemokines including *Ccl2*, *Ccl4*, *Ccl6*, *Ccl8*, *Ccl9* and *Osm* were upregulated in monocyte/macrophages of *KPC*-*Cdh11^+/+^* mice, suggesting possible candidates that can be therapeutically targeted to reduce the immunosuppressive nature and improve efficacy of existing therapeutics against PDAC. Inhibition of CCL2-CCR2 signaling has previously been shown to block the recruitment of inflammatory immune cells to the TME and inhibit cancer cell metastasis (56). Additionally, it has been suggested that *CCL4* can promote tumor development and progression by recruiting regulatory T cells and pro-tumorigenic macrophages and acting on other stromal cells present in the tumor microenvironment, such as fibroblasts and endothelial cells, to facilitate their pro-tumorigenic abilities (57). Ccl7 and Ccl9 may also facilitate tumor progression and metastasis (58, 59). *Cdh11* deficiency in the tumor microenvironment also altered the transcriptional profile of neutrophils. Neutrophils from *Cdh11*-deficient tumors had reduced expression of macrophage differentiation factor *Csf1* as well as several cytokines including *Ccl3*, *Ccl4*, *Ccl6*, *Il1b* and *Osm*. *Cdh11* deficiency in TME also contributed to downregulation of several cathepsins in monocyte/macrophages, neutrophils and DCs, many of which have already been shown to play a role in tumor progression and immune modulation (60). Future work aimed at therapeutically altering *Cdh11* and these cytokines and proteases may provide insight into successful approaches for modulating the immunosuppressive nature of the pancreatic TME.

These findings suggest that *Cdh11* deficiency in CAFs may alter the tumor immune microenvironment and contribute to an increased anti-tumor immune response. Our studies utilized mice that lack *Cdh11* since birth and therefore it is unknown if the lack of *Cdh11* from birth fundamentally changes fibroblast or any other *Cdh11*-expressing cells, priming an anti-tumor microenvironment. Further studies utilizing conditional knockouts and Cdh11 inhibition post-tumor formation will be required to confirm the role of CAF-derived Cdh11 in altering the tumor immune microenvironment.

We also observed low *Cdh11* expression in cancer cells undergoing EMT in both mice and human PDAC. Cdh11 has previously been shown to be associated with EMT in cancer (61) and antibody targeting of CDH11 inhibited EMT and suppressed metastasis in breast cancer (62). Interestingly, *KPC-Cdh11^+/+^* mice had significantly more EMT cells than *KPC-Cdh11^+/-^* mice. The reduced presence of EMT cells in *Cdh11*-deficient mice may have also contributed to the enhanced survival observed in these mice (23). In addition to PDAC, increased *Cdh11* expression was observed in many other cancers including breast cancer, stomach and colon cancer. These findings suggest that targeting *Cdh11* with small molecule inhibitors or function-blocking antibodies may be an effective strategy in treating aggressive tumors including PDACs.

*Cdh11* transcripts have been previously found in the peripheral blood as indicators of severe disease as in rheumatoid arthritis (63). The increased presence of *CDH11* in the peripheral blood of cancer patients may be indicative of an advanced disease state. The set of immune cell signatures identified in *Cdh11*-deficient mice may represent hallmarks of positive disease prognosis in pancreatic cancer, and maybe other solid tumors such as breast, head and neck and colorectal cancers. Targeting these specific immune cell subtypes or genes differentially expressed in these immune subpopulations as a result of *Cdh11* deficiency may be an effective therapeutic strategy to treat *Cdh11*-expressing cancers and fibrotic disease.

## Data availability statement

The datasets presented in this study can be found in online repositories. The names of the repository/repositories and accession number(s) can be found below: https://datadryad.org/stash, doi:10.5061/dryad.gxd2547s0.

## Ethics statement

The animal study was approved by Lawrence Livermore National Laboratory and GU IACUC. The study was conducted in accordance with the local legislation and institutional requirements.

## Author contributions

AS: Conceptualization, Data curation, Formal Analysis, Funding acquisition, Investigation, Methodology, Project administration, Supervision, Visualization, Writing – original draft, Writing – review and editing. KAM: Data curation, Formal Analysis, Funding acquisition, Investigation, Methodology, Validation, Visualization, Writing – review and editing. IP: Data curation, Formal Analysis, Investigation, Methodology, Resources, Validation, Visualization, Writing – review and editing. NRH: Investigation, Methodology, Writing – review and editing, Writing – original draft. NFL: Methodology, Validation, Visualization, Writing – review and editing. BA: Methodology, Validation, Visualization, Writing – review and editing. SPW: Data curation, Formal Analysis, Methodology, Validation, Visualization, Writing – review and editing. MAC: Funding acquisition, Project administration, Resources, Supervision, Writing – review and editing. EKW: Funding acquisition, Project administration, Supervision, Writing – review and editing. SWB: Conceptualization, Funding acquisition, Methodology, Project administration, Resources, Supervision, Writing – review and editing. GGL: Conceptualization, Funding acquisition, Project administration, Resources, Supervision, Visualization, Writing – review and editing.
