# Supplementary material for: Loss of Cadherin-11 in pancreatic ductal adenocarcinoma alters tumor-immune microenvironment
Source: Front Oncol. 2023 Oct 26;13:1286861. doi: 10.3389/fonc.2023.1286861 (PMC10639148; doi:10.3389/fonc.2023.1286861)
Supplement: Supplementary file 1 [file DataSheet_1.pdf]

Supplementary Material

Loss of Cadherin-11 in pancreatic ductal adenocarcinoma alters tumor-immune microenvironment

Aimy Sebastian[1#], Kelly A. Martin[1#], Ivana Peran[2], Nicholas R. Hum[1], Nicole F. Leon[1], Beheshta Amiri[1], Stephen P. Wilson[1], Matthew A. Coleman[1], Elizabeth K. Wheeler[1], Stephen Byers[2], Gabriela G. Loots[1,3]

[1]Lawrence Livermore National Laboratory, Physical and Life Science Directorate, Livermore, CA.

[2]Georgetown-Lombardi Comprehensive Cancer Center, Department of Oncology, Georgetown University Medical Center, Washington, DC, USA

[3]University of California Davis Health, Department of Orthopaedic Surgery, Sacramento, CA.

# Authors contributed equally to the manuscript

*** Correspondence:**
sebastian4@llnl.gov

gloots@ucdavis.edu

Supplementary Figures

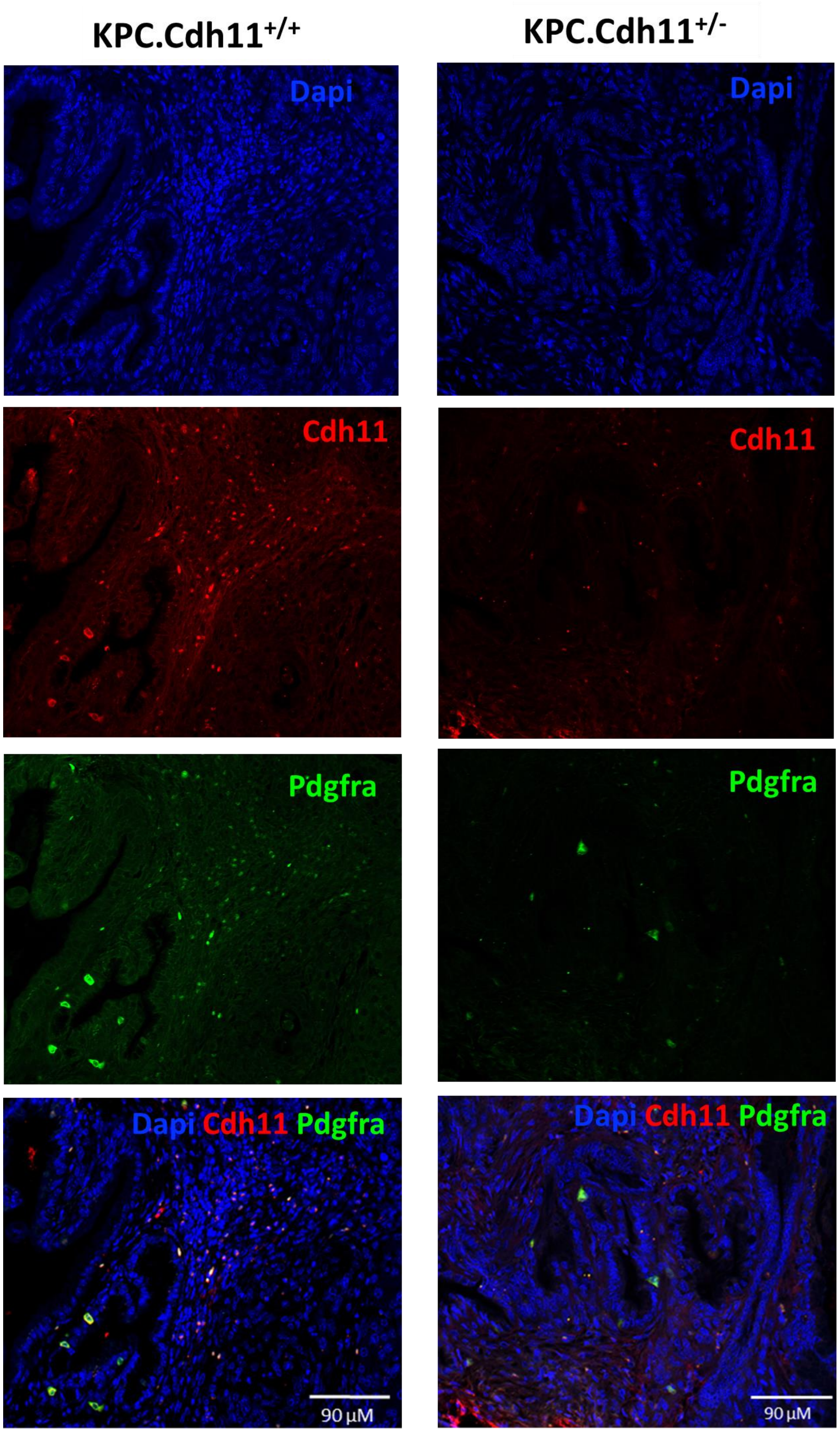

Figure S1: Immunohistochemistry analysis of Cdh11 expression. CAFs from KPC-Cdh11$^{+/+}$ mice co-expressed CAF marker *Pdgfra* and *Cdh11*.

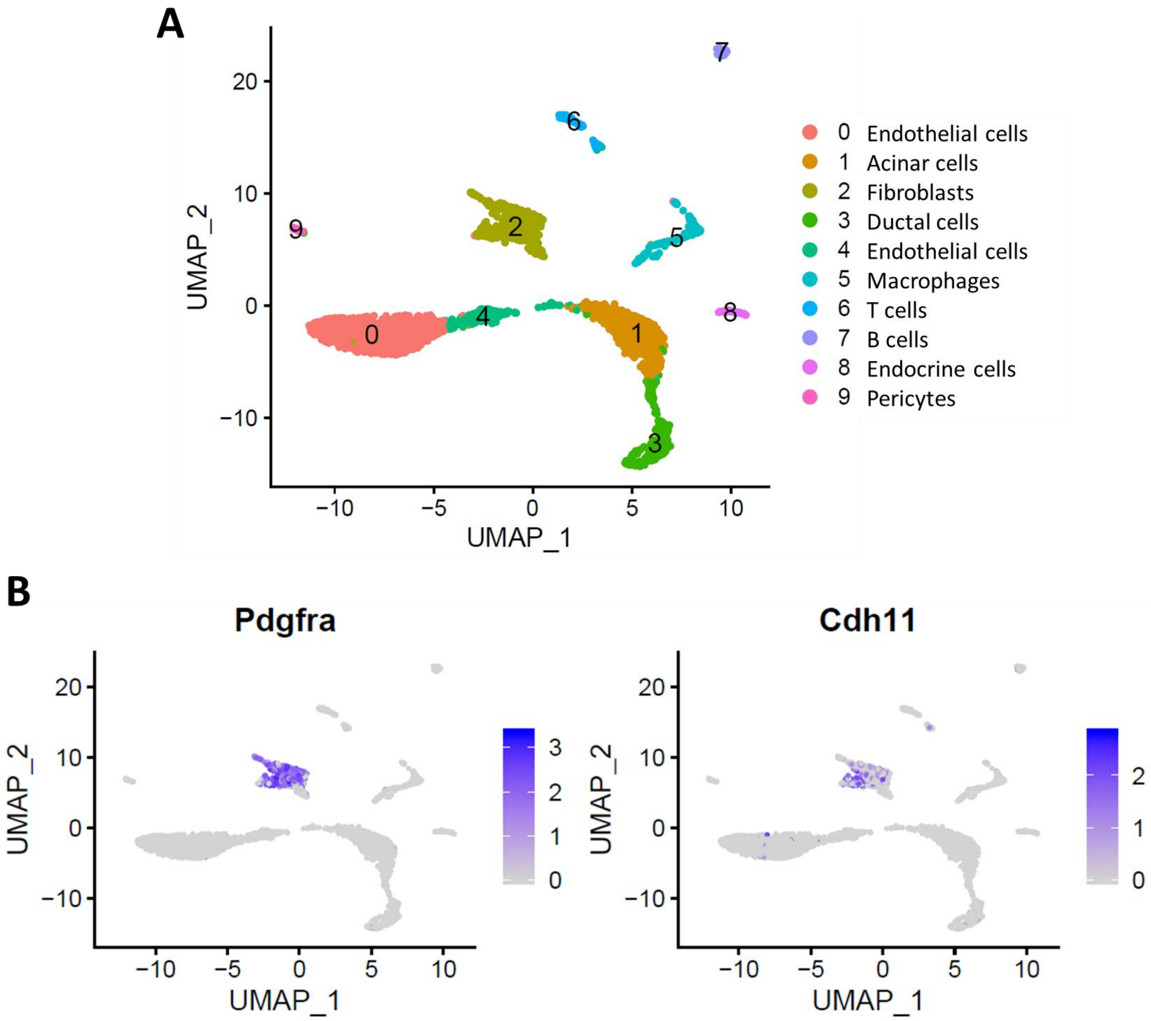

Figure S2: **Cdh11 expression in pancreas from non-tumor bearing mice.** A) UMAP plot showing cell types identified in pancreas from non-tumor bearing wildtype mice. B) *Cdh11* and *Pdgfra* expression in normal fibroblasts from non-tumor bearing mice.

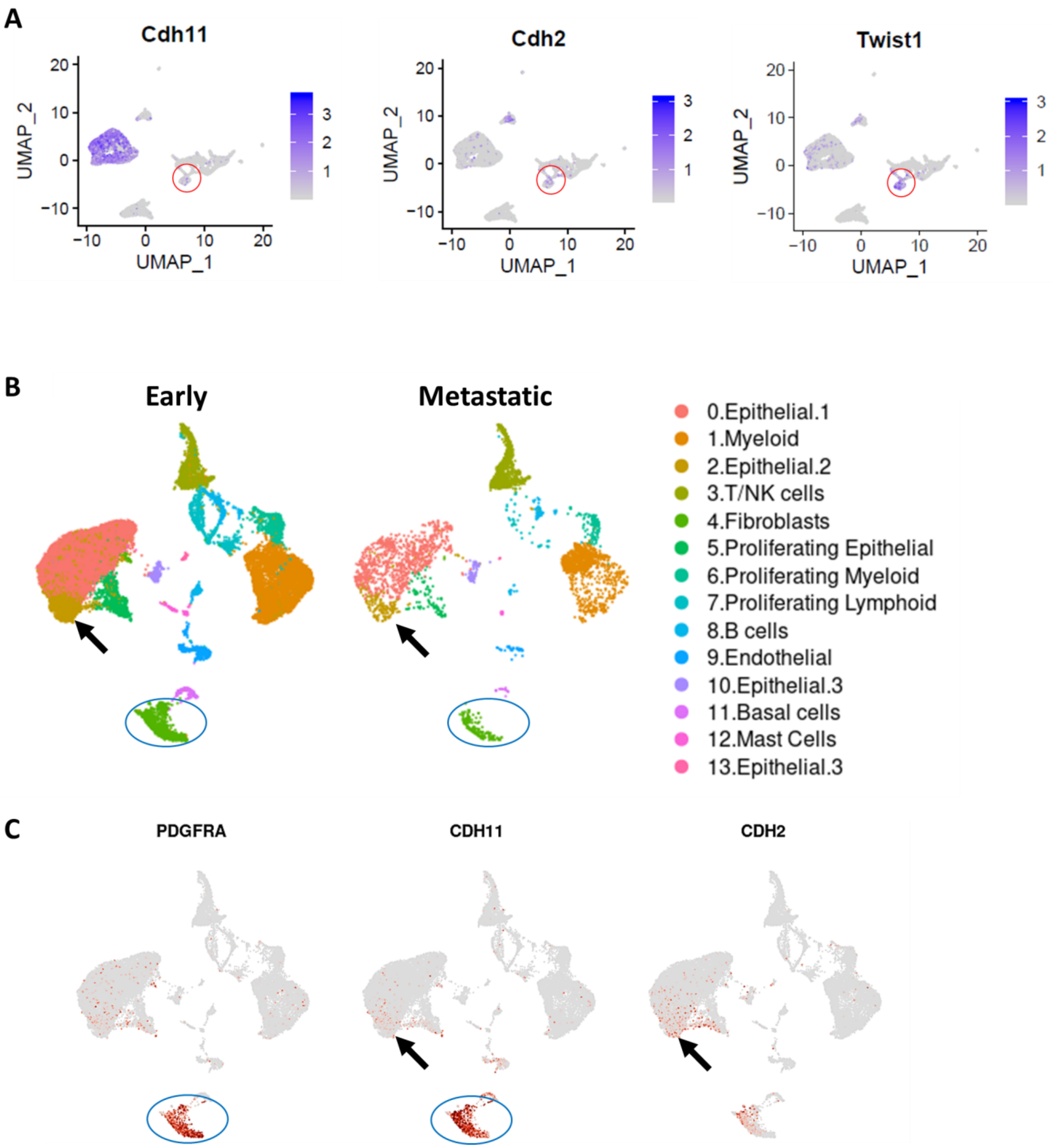

Figure S3. ***Cdh11* expression in EMT cells.** A) EMT cells (red oval) in KPC mice expressed low levels of *Cdh11* along with EMT markers *Cdh2* and *Twist1*. B) UMAP plot of cells from early and metastatic human PDACs. Blue oval shows CAFs and black arrow shows EMT cells expressing *CDH2*. C) *CDH11* expression (red) in CAFs and EMT cells from early and metastatic human PDACs.

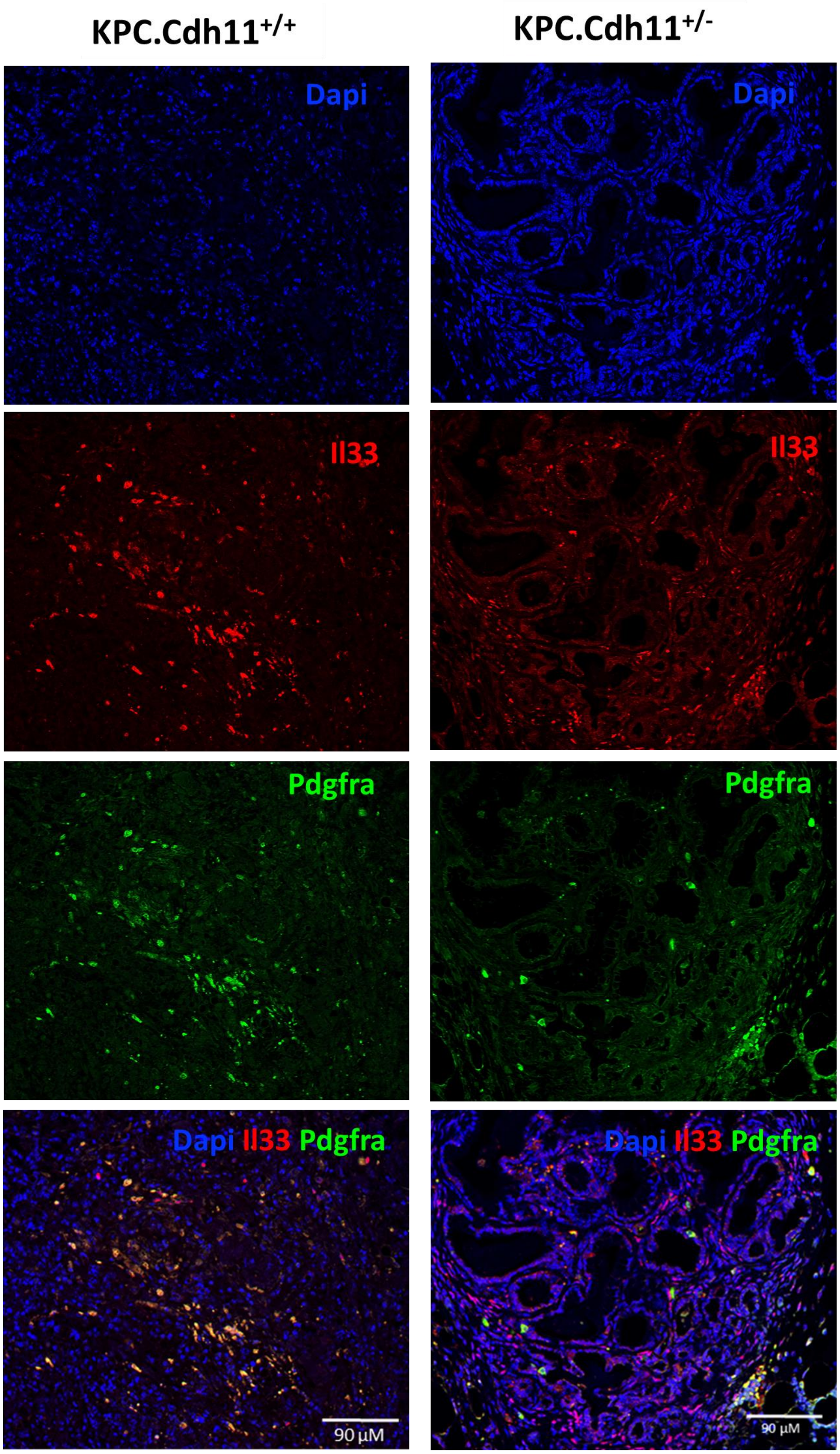

Figure S4. Il33 expression in CAFs. IHC analysis showed an increased number of Il33-expressing CAFs (Pdgfra+ cells) in *KPC-Cdh11*$^{+/+}$ pancreas compared to *KPC-Cdh11*$^{+/-}$ pancreas.

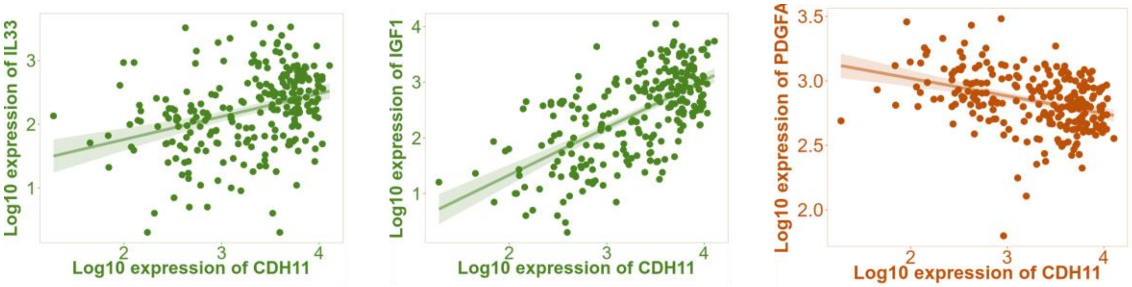

Figure S5. **Correlation between the expression of *Cdh11* and other genes in human PDAC.** Correlations between the genes were determined using TNMplot.

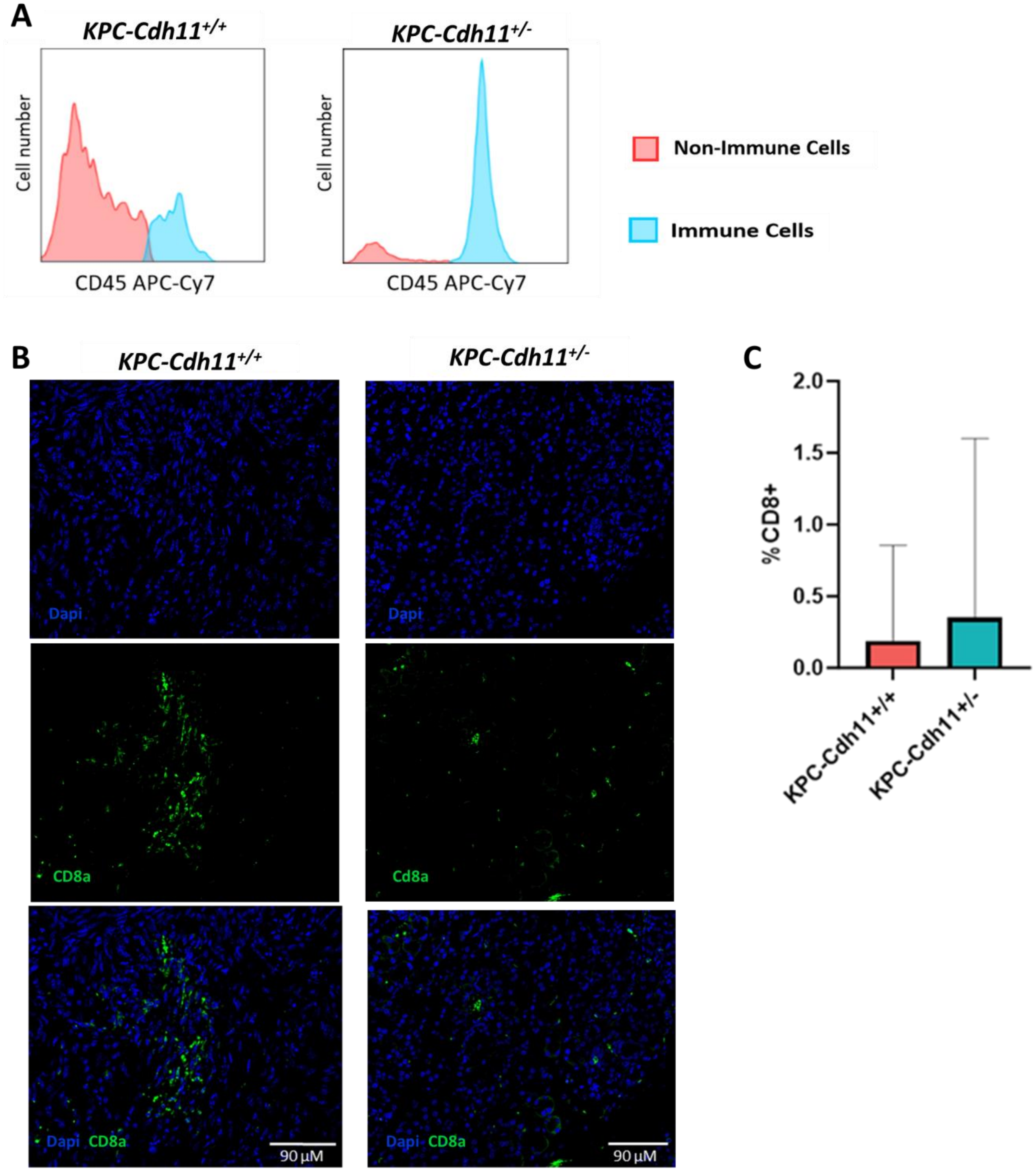

Figure S6. **Immune cells in tumor microenvironment.** A) FACS analysis of cells pooled from five *KPC-Cdh11*$^{+/+}$ and *KPC-Cdh11*$^{+/-}$ pancreases showed that *Cdh11*-deficient tumors have increased immune infiltration. B) IHC images showing CD8a expression in *KPC-Cdh11*$^{+/+}$ and *KPC-Cdh11*$^{+/-}$ pancreases. C) Quantification of CD8a staining of *KPC-Cdh11*$^{+/+}$ and *KPC-Cdh11*$^{+/-}$ pancreases using Vectra quantitative pathology imaging system.

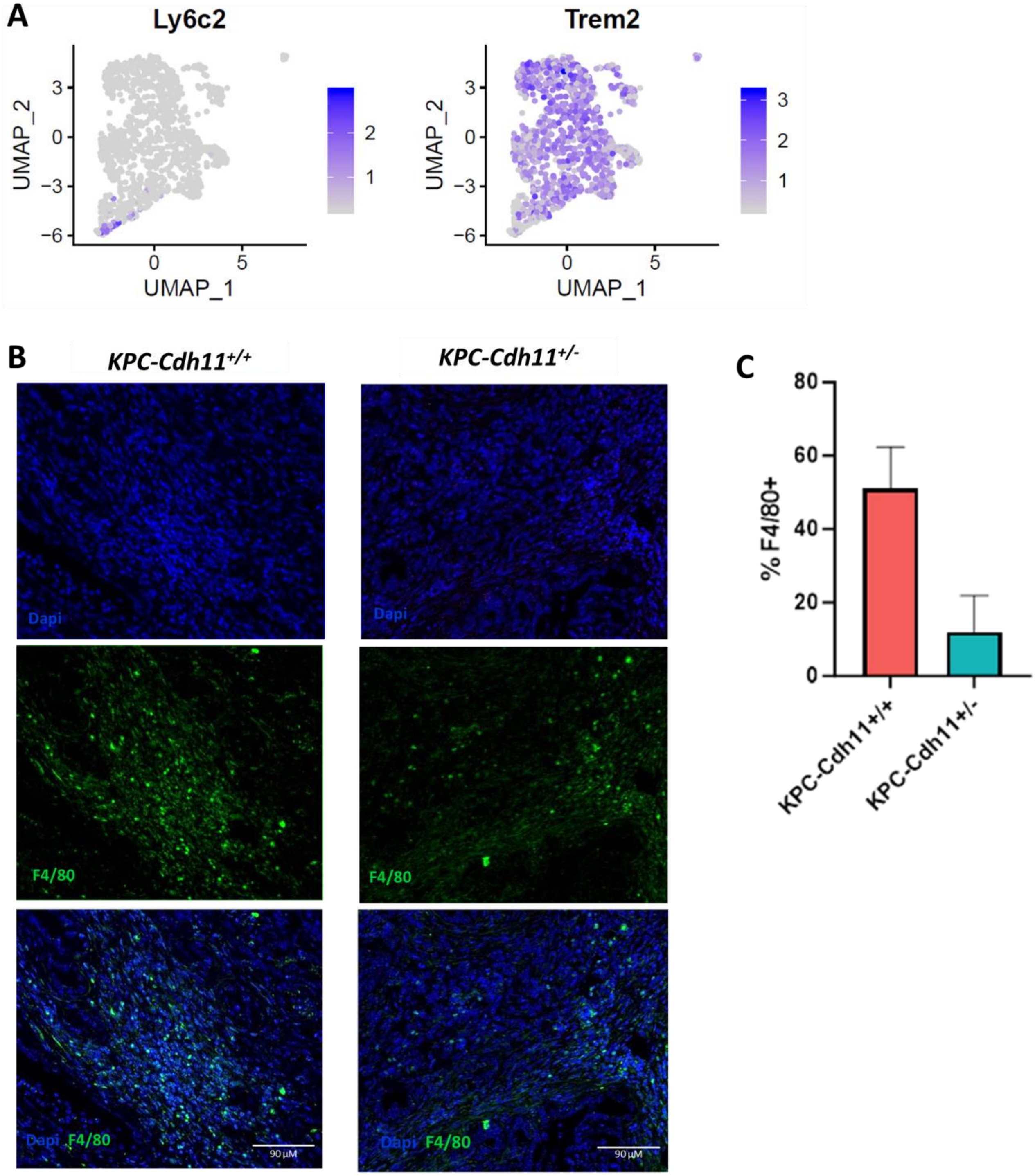

Figure S7. **Macrophages in PDAC tumor microenvironment.** A) *Ly6c2* and *Trem2* gene expression (blue) in Mono-Mac clusters. B) IHC images showing F4/80 expression in *KPC-Cdh11*$^{+/+}$ and *KPC-Cdh11*$^{+/-}$ pancreases. C) Quantification of F4/80^{+} staining of *KPC-Cdh11*$^{+/+}$ and *KPC-Cdh11*$^{+/-}$ pancreases.

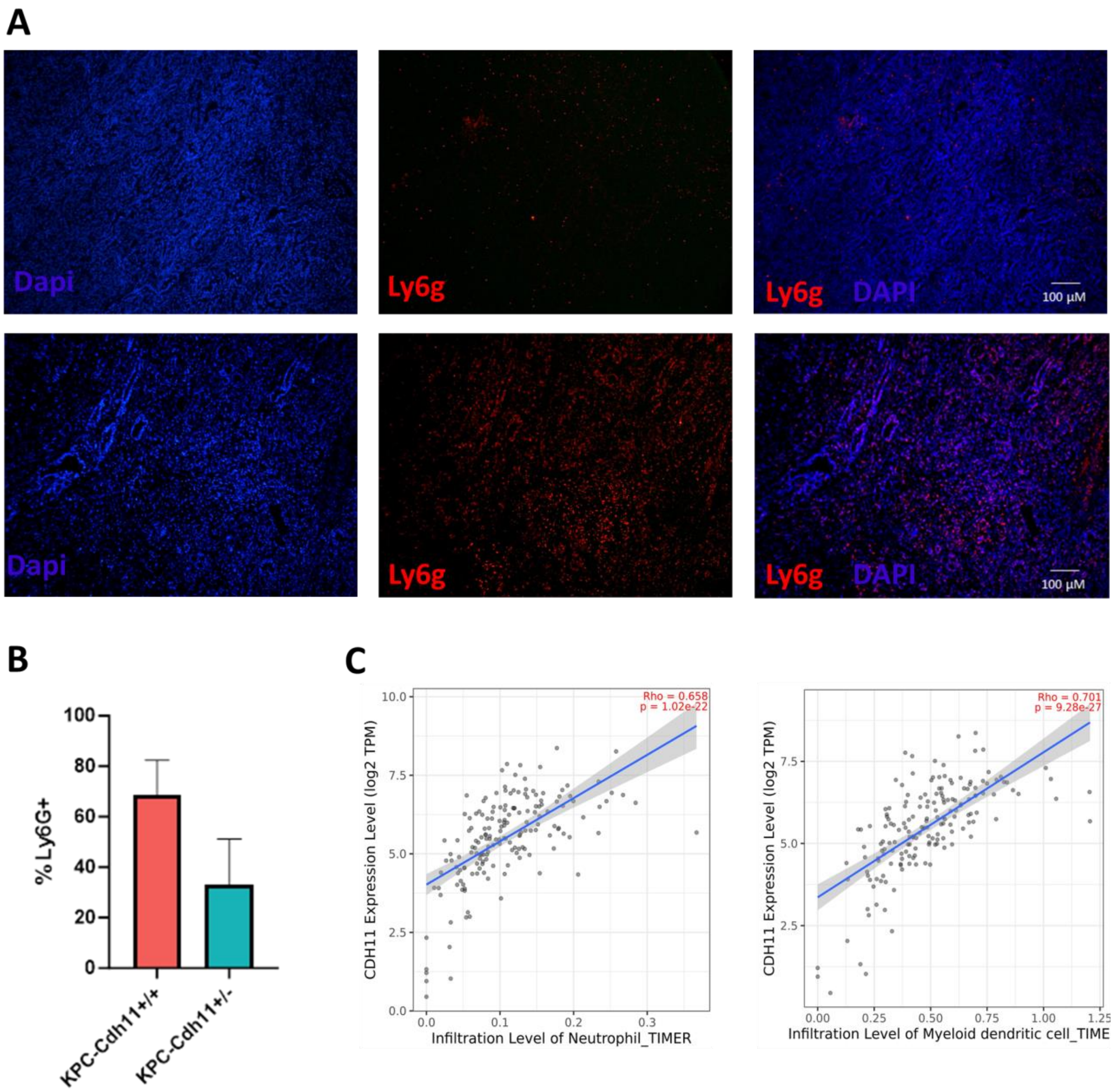

Figure S8. **Neutrophils and other myeloid cells in PDAC tumor microenvironment. A)** IHC images showing Ly6g expression in *KPC-Cdh11*$^{+/+}$ and *KPC-Cdh11*$^{+/-}$ pancreases. B) Quantification of Ly6g staining of *KPC-Cdh11*$^{+/+}$ and *KPC-Cdh11*$^{+/-}$ pancreases. C) Correlation between *Cdh11* expression and neutrophil and DC infiltration in human PDAC, identified using TIMER.

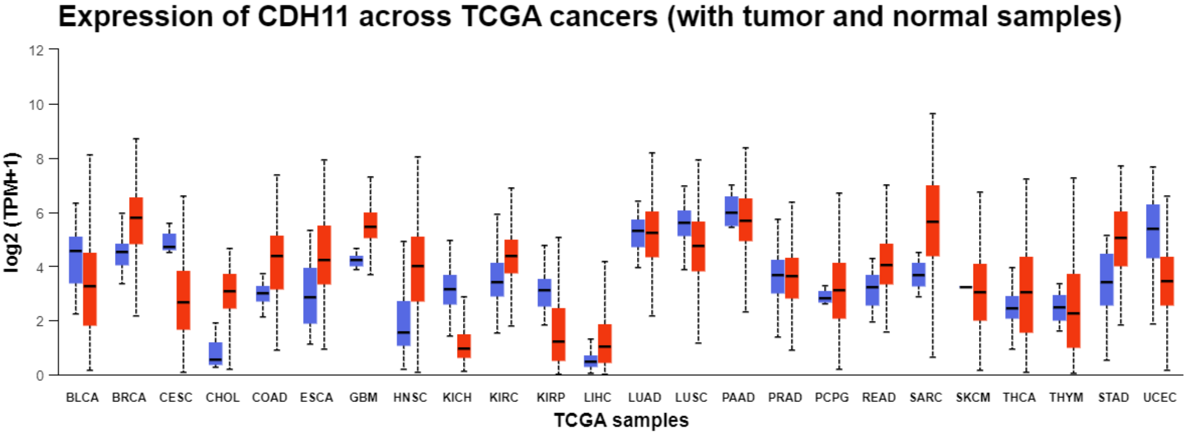

Figure S9. **CDH11 expression across various human tumors.** Data obtained from UALCAN.
